# COVID-19-Associated Multisystem Inflammatory Syndrome Complicated with Giant Coronary Artery Aneurysm

**DOI:** 10.1155/2021/8836403

**Published:** 2021-01-06

**Authors:** Mohammad Reza Navaeifar, Leila Shahbaznejad, Ali Sadeghi Lotfabadi, Mohammad Sadegh Rezai

**Affiliations:** ^1^Pediatric Infectious Diseases Research Center, Communicable Diseases Institute, Mazandaran University of Medical Sciences, Sari, Iran; ^2^Department of Pediatrics, BuAli Sina Hospital, Faculty of Medicine, Mazandaran University of Medical Sciences, Sari, Iran

## Abstract

In the early stages of the outbreak of the novel coronavirus disease 2019 (COVID-19), it was assumed that this infection is very mild and uncommon in children. However, recent reports have shown that children may also develop the disease and its severe complications. These complications included shock, multisystem inflammatory syndrome in children (MIS-C), and pneumonia in children. A previously healthy 14-month-old boy presented with fever, irritability, and skin rash, besides changes in the lips, conjunctiva, and tongue. His medical history, clinical presentations, treatment, laboratory data, and follow-up information were recorded. He was treated according to the diagnosis of Kawasaki disease (KD). He had a history of close contact with a COVID-19 patient. However, the result of reverse transcription-polymerase chain reaction (RT-PCR) assay for COVID-19 was negative. Immunoglobulin M for COVID-19 was positive (1.20), while immunoglobulin G was negative (0.37). Three weeks later, seroconversion of COVID-19 immunoglobulin G (1.42) occurred. Despite treatment with two doses of intravenous immunoglobulin and methylprednisolone, coronary artery ectasia was detected. On the sixth day of hospitalization, the patient experienced hypotension, which necessitated treatment with inotropic drugs and resulted in a change of diagnosis to MIS-C. The later echocardiography showed evidence of coronary artery aneurysm (CAA), which finally changed to giant CAA. Although the patient was treated with infliximab, the size of CAA showed a significant decrease in the one-month follow-up. This is the first report of MIS-C during the COVID-19 pandemic in Iran, accompanied by KD, which was complicated with giant CAA.

## 1. Introduction

In December 2019, the outbreak of a novel coronavirus disease (COVID-19) was reported in Hubei Province, China. The rapid spread of the disease and the high number of patients affected many countries around the world, and the disease was declared as a global pandemic by the World Health Organization (WHO) on March 11, 2020. Considering the spread of this virus around the world, many studies have focused on the symptoms and side effects of COVID-19. Although the disease was thought to be very mild and uncommon in children for a short period [1, 2], recent reports have shown that children may also develop the disease and its complications [3–5]. To date, there have been reports of symptoms, such as rash, shock, severe systemic inflammation, and pneumonia in children, associated with COVID-19 [6].

It is important to pay particular attention to systemic problems during the COVID-19 pandemic such as multisystem inflammatory syndrome in children (MIS-C) [7, 8]. Although the pediatric multisystem inflammatory syndrome (PIMS) has been reported in children as one of the complications of COVID-19, there are some concerns about missing the diagnosis of Kawasaki disease (KD), an inflammatory disease of childhood, which has been linked to some viruses and may lead to shock and coronary artery aneurysm (CAA) [9, 10].

However, giant CAA has been rarely detected in COVID-19 patients [6, 11, 12]. Here, we present the case of a child with MIS-C, accompanied by KD following the COVID-19 epidemic, complicated with giant CAA.

## 2. Case Presentation

The patient was a previously healthy 14-month-old boy (weight, 12 kg; height, 77 cm), presenting with fever, irritability for five days, and skin rash for three days before admission. The erythematous rash first began in the trunk and upper limbs and then spread to the hands and feet, accompanied by the swelling of the extremities. Changes in the lips and tongue were also observed.

During this period of the disease, he just received azithromycin and acetaminophen. His father was the COVID-19 index case, and they had close household contact while the father had cough before COVID-19 diagnosis, seven days before the child's symptoms began.

Upon admission to Bu-Ali Sina Hospital in Sari, north of Iran, in April 2020, the patient was irritable and ill. Physical examination revealed fever (38.8°C), respiratory rate of 32 bpm, pulse rate of 120 bpm, and blood pressure of 84/42 mmHg. He had cracked and erythematous lips, a strawberry tongue, and bilateral nonpurulent conjunctivitis. A generalized erythematous maculopapular rash was also observed on the skin. The heart, lung, and abdominal findings were unremarkable on the physical examination. Besides the rash, edema was detected on the upper and lower extremities. There was no significant cervical lymphadenopathy.

According to the definition of KD by the American Heart Association [13], the patient was hospitalized with an initial diagnosis of KD. In the primary laboratory test, he showed leukocytosis, neutrophilia, anemia, elevated erythrocyte sedimentation rate (ESR), elevated C-reactive protein (CRP), and increased liver transaminase levels. The real-time reverse transcription-polymerase chain reaction (RT-PCR) assay for COVID-19 was negative in the nasopharyngeal specimen. The serum albumin level was decreased (2.3 g/dL), and the serum 25-hydroxyvitamin D level was 33 ng/mL (normal range). Also, the kidney function, coagulation tests, and lactate dehydrogenase (LDH) were in the normal range, and the first chest X-ray was unremarkable (Table 1).

On the second day, an echocardiography was performed, and the results are reported in Table 1. The chest sonography revealed minimal (5 mm) right-sided pleural effusion.

Three days later, the patient was still febrile. He became toxic, tachypneic (respiration rate, 44 bpm), and much irritable and showed mild abdominal distention without significant hepatomegaly. Consequently, he was transferred to the pediatric intensive care unit (PICU). His pulse rate was 165 bpm, blood pressure was 78/42 mmHg, oxygen saturation was 89% (based on pulse oximetry), and the extremities were cold with a capillary refill time of three seconds.

In new laboratory data, the serum immunoglobulin M antibody for COVID-19 (measured using microliter wells coated with SARS-CoV-2 nucleocapside antigen kit, Pishtazteb Company) reported positive (1.20), and the immunoglobulin G antibody was negative (0.37). Prothrombin time was 13 seconds, and partial thromboplastin time was 60 seconds.

**Figure 1:**
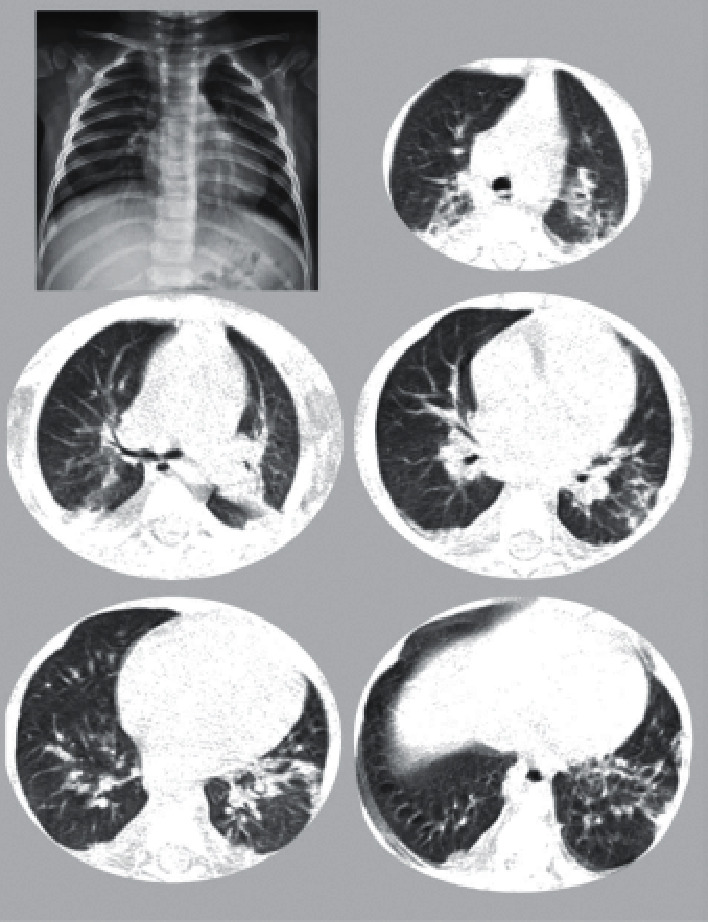
Chest X-ray and CT scan on the 3^rd^ day of admission show bilateral ground-glass opacities.

The changes in the patient's condition encouraged the physicians to review his clinical status, and a diagnosis of MIS-C, accompanied by KD following the COVID-19 epidemic, was established. The second echocardiography indicated mild dilation of coronary arteries, without decreased left ventricular ejection fraction (LVEF). Although the first-day chest CT scan showed mild nonspecific interstitial changes, the second CT scan on the third day demonstrated bilateral ground-glass opacities with consolidation and the halo sign (Figure 1). Persistent fever and deterioration of the patient's condition necessitated the administration of the second dose of IVIG. Moreover, he required packed red blood cells to correct anemia.

Six days after admission, the patient's condition worsened and he became hypotensive, while the fever continued and his abdominal distention got worse. The liver and spleen were mildly enlarged, and there was mild free fluid in abdominal ultrasonography.

Over the following days, inotropic drugs were gradually discontinued and prednisolone was tapered. Also, clopidogrel was added to ASA for the prophylaxis of CAA thrombosis.

**Figure 2:**
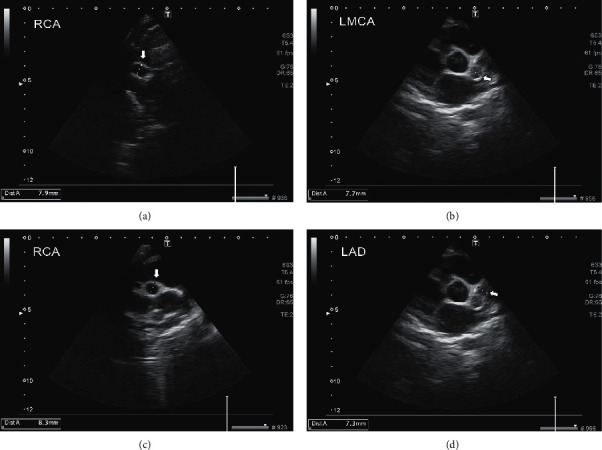
Echocardiography on the 20^th^ day of admission shows giant aneurysms in coronary arteries. (a) RCA, (b) LMCA, (c) RCA, and (d) LAD.

On the 14^th^ day, when rash and tachypnea were improved, there was a slight peeling on the extremities, the patient looked well, and the platelet count continued to rise. The echocardiography showed giant coronary artery aneurysms (*z*-score for all three main coronary arteries >10, see Table 1), and it was necessary to add heparin infusion to the previous antiplatelet drugs. Moreover, one dose of infliximab was administered (Figure 2).

On the 20^th^ day, the patient was transferred to the ward, and on the 24^th^ day, he was discharged and recommended to take antiplatelet drugs and warfarin for thromboprophylaxis. Three weeks later, seroconversion of COVID-19 immunoglobulin G from negative (0.37) to positive (1.46) occurred, while immunoglobulin M antibody remained positive. The one-month follow-up after discharge showed that the coronary arteries were still aneurysmal but had significantly reduced in size.

A written informed consent was obtained from the patient's parents for publication of his information and medical images. This report could contribute to the study of clinical and paraclinical characteristics of COVID-19 and was approved by the university ethical committee (ethical code: IR.MAZUMS.REC.1398.7277).

## 3. Discussion

In this report, we presented a case of COVID-19-associated MIS-C, complicated with giant CAA. The diagnosis of COVID-19 was initially made, based on the patient's history of contact with a known case of COVID-19, which was finally confirmed by seroconversion for immunoglobulin G antibody.

COVID-19 is a new disease with a variety of clinical manifestations, which have been identified since its emergence. Also, the manifestations of this disease are diverse in children [6, 7, 9, 13–15].

Multisystem inflammatory response and other manifestations of COVID-19 such as shock, rash, edema, and hypoalbuminemia can lead to miss concepts in detection and treatment of KD [16]. Delayed appropriate management can lead to CAA in up to 25% of KD cases and potential mortality [17–19]. This potential diagnostic delay has raised some concerns about the timely diagnosis and treatment of KD during the COVID-19 pandemic.

Different types of coronary artery involvement may occur in KD, coronary artery dilation may be seen in up to 50% of cases, and 0.7–7% may lead to CAA, especially in younger patients [17, 19].

A recent study by Whittaker et al. reported two giant CAAs (*z*-score >10) in children with multiple systemic inflammations during the COVID-19 pandemic. The involvement of coronary arteries in COVID-19-associated MIS-C patients, even in the absence of typical criteria for KD, indicates the need for more attention to these vessels, especially if the patient has symptoms of shock or severe systemic inflammation [20].

Although myocardial infarction has been reported in about 18% of KD cases, one of the major concerns is that a giant CAA may be asymptomatic until ischemia develops [21].

As a risk factor of the formation of CAA, our patient was a young child near infancy complicated with shock and was resistant to initial IVIG. Whether or not COVID-19 is a risk factor for CAA is still unclear and requires further study.

Although early IVIG therapy can reduce the risk of CAA, CAA and giant CAA may still occur in 5% and 1% of cases, respectively [18].

The use of an anti-TNF-*α* monoclonal antibody as adjuvant therapy in IVIG-resistant KD reduces the size of CAA [21, 22]. Although it is not yet possible to comment definitively on the use of these drugs in COVID-19 in children and adults, they can inhibit fusion of the new coronavirus with human cells and decrease the mortality rate [23, 24].

This is the first report of MIS-C, accompanied by KD during the COVID-19 pandemic in Iran, which was complicated with giant CAA.

## Figures and Tables

**Table 1:** The main clinical course, medications, results of laboratory tests, and changes in the patient's cardiac involvement during the hospital stay.

Variable	Hospital days					
	1^st^	3^rd^	6^th^	10^th^	14^th^	20^th^
General appearance	Ill	Toxic	Toxic	Mildly ill	Fair	Good

Fever	Yes	Yes	Yes	No	No	No

Rash	Yes	Yes	Yes	Yes	No	No

Tachypnea	No	Yes	Yes	Yes	No	No

Hypotension	No	No	Yes	No	No	No

Total white blood cell count, ×10^6^/L	22,000	21,800	27,000	28,300	25,700	21,880

Neutrophil, %	83	79	79	72	38	57

Lymphocyte, %	5	15	10	11	44	30

Monocyte, %	3	1	10	14	17	7

Hemoglobin, g/L	10.6	8.7	10	11.9	11.7	11.4

Platelet count, ×10^6^/L	197,000	224,000	420,000	863,000	1,168,000	964,000

ESR	65	—	75	72	25	3

CRP, mg/L	38	—	38	25	10	29

AST, mg/dl	200	55	42	—	38	—

ALT, mg/dl	197	57	35	—	32	—

Antibiotic(s)	Cefotaxime	Meropenem, vancomycin	Meropenem, vancomycin	Meropenem	Meropenem	—

Daily main changes in medications	(i) IVIG (1^st^) 2 gr/kg (ii) ASA 15 mg/kg/q6h (iii) Albumin 1 g/kg/day for 3 days (iv) Hydroxychloroquine 5 mg/kg/day for 5 days	(i) IVIG (2^nd^) 2 gr/kg (ii) Packed RBC transfusion 10 ml/kg (iii) Captopril 0.3 mg/kg/q8h for 3 days (iv) Fresh frozen plasma 10 ml/kg	(i) Prednisolone 2 mg/kg/day (ii) Dopamine 6 micg/kg/min (iii) Milrinone 0.5 micg/kg/min (iv) Packed RBC transfusion 10 ml/kg (v) Albumin 1 g/kg/day for 3 days	(i) Clopidogrel 0.5 mg/kg/day continued until discharge (ii) Captopril 0.3 mg/kg/q8h	(i) Prednisolone tapered (ii) Heparin 10–20 U/kg/h for 3 days (iii) ASA 5 mg/kg/day continued until discharge	(i) Warfarin 0.1 mg/kg/day started from the 16^th^ day and continued until discharge (ii) Infliximab 5 mg/kg on the 19^th^ day

Echocardiography	Minimal MR, normal coronary arteries, good LVEF	Mild to moderate MR, TR and PI, mild coronary dilatation in LAD and LCX, diastolic dysfunction, good LVEF	Moderate MR and TR, ectatic coronary arteries, mild diastolic dysfunction, LVEF: 43%	Minimal MR, mild TR and PI, moderate aneurysmal coronary artery: RCA: 5.8 mm, LMCA: 6.2, LAD: 5.9, LVEF: 62%	Minimal MR, mild TR, minimal PI, dilated aneurysmal RCA: 7.8 mm, LMCA: 7.7 mm, LAD: 5.7 mm, good LVEF	Minimal MR, no TR, minimal PI, giant aneurysms in RCA: 8.3 mm, LMCA: 7.7 mm, LAD: 7.3 mm, good LVEF

ESR = erythrocyte sedimentation rate; CRP = C-reactive protein; ALT = alanine aminotransferase; AST = aspartate aminotransferase; MR = mitral regurgitation; TR = tricuspid regurgitation;PI = pulmonary insufficiency; PE = pericardial effusion; RCA = right coronary artery; LAD = left anterior descending coronary artery; LMCA = left main coronary artery; LVEF = left ventricular ejection fraction.

## Data Availability

The case's medical data are scanned and stored in our medical data center and are available upon request.
